# Competition-Colonization Trade-Offs, Competitive Uncertainty, and the Evolutionary Assembly of Species

**DOI:** 10.1371/journal.pone.0033566

**Published:** 2012-03-20

**Authors:** Pradeep Pillai, Frédéric Guichard

**Affiliations:** Department of Biology, McGill University, Montreal, Quebec, Canada; CNRS, University of Montpellier II, France

## Abstract

We utilize a standard competition-colonization metapopulation model in order to study the evolutionary assembly of species. Based on earlier work showing how models assuming strict competitive hierarchies will likely lead to runaway evolution and self-extinction for all species, we adopt a continuous competition function that allows for levels of uncertainty in the outcome of competition. We then, by extending the standard patch-dynamic metapopulation model in order to include evolutionary dynamics, allow for the coevolution of species into stable communities composed of species with distinct limiting similarities. Runaway evolution towards stochastic extinction then becomes a limiting case controlled by the level of competitive uncertainty. We demonstrate how intermediate competitive uncertainty maximizes the equilibrium species richness as well as maximizes the adaptive radiation and self-assembly of species under adaptive dynamics with mutations of non-negligible size. By reconciling competition-colonization tradeoff theory with co-evolutionary dynamics, our results reveal the importance of intermediate levels of competitive uncertainty for the evolutionary assembly of species.

## Introduction

Underlying various evolutionary models of community assembly have been different ecological mechanisms of species coexistence. Most often evolutionary models of community assembly assume coexistence results from the operation of resource partitioning mechanisms [1] that minimize niche overlap between competitors. As such, the evolutionary assembly of communities arises when character displacement in the traits associated with resource use allows the stabilization of competitive interactions between species within an assemblage [2]–[8].

An alternative ecological mechanism for species coexistence involves life-history trade-offs between competitive ability and fecundity (or mortality) [9]–[12]. This coexistence mechanism, however, has proved a more problematic basis for modelling the evolution of adaptive communities. Classic metapopulation models based on a strict competition-colonization tradeoffs, and spatial subdivision of habitat resources, have – despite their widespread use in explaining biodiversity *maintenance* – been problematic for studying the evolutionary *emergence* or assembly of communities due to their inability to predict stable multispecies communities, or the evolutionary build-up of biodiversity [13]. Here we study competitive uncertainty through the use of a competition function introduced by Calcagno et al [14] to study multispecies coexistence. Using this framework, we show how current metapopulation models can be used as a framework for the study of evolutionary assembly of species.

Multi-species metapopulation – or metacommunity – models [10], [12] describe competitive interactions as being equally strong between species regardless of the difference in trait value. Since some trait value is traded off with competitive ability, species with a more advantageous trait value (e.g. higher fecundity or lower mortality) will be competitively excluded by species with a less advantageous trait value in local interactions. Coexistence becomes possible by the spatial subdivision of the habitat, which allows persistence of the poorer competitors at the regional spatial scale despite exclusion within local sites, because of the compensating effects of such traits as higher fecundity or lower mortality [9], [11], [12]. Examples include predator-induced mortality among marine mollusks [15], competition for habitat among African acacia-ants [16] or insects spatially partitioning food resource patches [17], [18].

Competitive life-history trade-offs have been incorporated into several evolutionary studies that involve mechanistic models of resource competition and niche overlap [19], [20]. However, efforts to utilize standard metapopulation models as an evolutionary framework for studying species assembly in regional communities, or *metacommunities*, have faced a number of challenges related to predictions of unrealistic adaptive or co-evolutionary dynamics. More precisely, metapopulation models with strict competitive hierarchies have been shown capable of allowing an infinite number of species to pack into the system at the low abundance (high competitive) threshold of the trait gradient [13]. This prediction of infinite diversity in invasion-structured communities, combined with biologically unrealistic low abundances, poses a challenge to the use of such models when studying equilibrium community assemblages. One way to addressing this challenge is to relax the classic assumption of strict competitive hierarchies [10], [12].

In natural populations interactions between competitors are better described as being probabilistic in nature due to the random individual differences within species that often weaken the competitive advantage one species has over an inferior competitor [21]–[24]. Such competitive uncertainty between species has been associated with co-evolution and stable co-existence between two competitors [25], as well as speciation through deterministic branching in Lotka-Volterra and mechanistic competition models [19], [26]. Calcagno *et al.* [14] also studied how the introduction of competitive uncertainty could enable the coexistence of multiple species in competition-colonization trade-off models. Here we adopt the metapopulation model of Calcagno et al. in order to study the evolutionary assembly of whole metacommunities.

We first demonstrate the inability of the standard metapopulation model of Tilman [12] to allow for the evolution of a stable assemblage due to the evolutionary extinction of all species, as was suggested by the earlier results of Kinzig *et al.* [13]., We then analyze trait evolution in the metapopulation model of Calcagno et al. [14], where the strict nature of competitive exclusion has been relaxed, making the outcome of competitive interactions between two individuals of different species probabilistic. Studying the adaptive dynamics of this metapopulation model we report the evolution of stable multispecies assemblages, and also demonstrate how intermediate degrees of competitive uncertainty maximize the equilibrium species biodiversity, as well as the possibility that biodiversity could emerge through species diversification and adaptive radiation in the presence of transient phenotypes arising from mutations of non-negligible sizes.

## Results

### Metacommunity dynamics

The dynamics of the regional community – or metacommunity – are given by the metapopulation equations for multiple competing species [9], [10], [12], [27]. The metapopulation formalism we adopt here is interpreted as applying at the scale of individuals that are colonizing and occupying microsites in a spatially implicit habitat landscape [10], [12]. In this model the community is structured by a competitive-colonization trade-off, where a transitive hierarchy for competitive ability exists amongst species such that species *1*>species *2*>**…** species *i* **…**>species *n*. The model further assumes that lower ranking species are always capable of displacing or overgrowing species of a higher rank index, while species of higher rank can never displace species of lower rank. As part of the trade-off a transitive hierarchy for colonizing ability also exists but in the opposite direction such that species *1*<species *2*<… species *i* …<species *n*. Each species *i* has an abundance *p_i_*, representing the fraction of the total landscape that is occupied by the species. The rate of change in the proportion of sites occupied by the *i*th species can thus be represented by the following equation [12]:
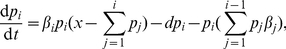
(1)where *β_i_* represents the colonizing rate of the *i*th species and 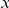 represents the proportion of the total landscape available for colonization. Mortality for all species is represented by *d*. In the first term 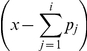 represents the proportion of sites available for colonization by the *i* ^th^ species. The third term gives the loss due to competitive displacement by the spread of superior competitor species (*j*<*i*) into sites occupied by *i*.

The minimum colonization rate 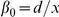 required for persistence on the landscape is found by solving for *β*_1_ when 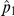 is set to zero. For any species *i* to persist in the landscape its colonization rate must be larger than this minimum threshold (i.e. *β_i_*>*β*_0_).

#### Evolutionary dynamics

**Figure 1:**
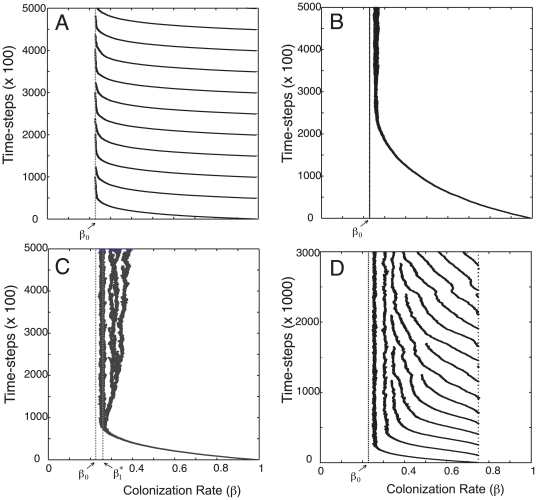
Time-series of stochastic simulations depicting evolution of species. In all simulations species were introduced at the high colonization (low competitive) end of trait space and allowed to subsequently evolve For all simulations shown *d* = 0.6, 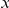 = 0.654, and the average mutation in trait value *β*, for every bout of reproduction, is *μ* = 0.001* (1/N) unless otherwise stated (see Methods for details). (A) Simulation when community is defined by classic competition-colonization trade-off of Equation (1) with the minimum threshold, *β*_0_, indicated by the vertical dashed line. (B) Evolutionary dynamics in a one-species system for the generalized metapopulation model of Equation (2) with *k* = 60. Species now avoid evolution to stochastic extinction by evolving to a singular strategy, *β*_1_^*^, some distance above the minimum threshold *β*_0_. (C) Illustration of disruptive selection with high average mutation distance (*μ* = 0.0095*(1/N)) in trait value and intermediate *k* values (*k* = 60). (D) Community assembly for a large number of species for *k* = 60 and with distinct phenotypic distances between strategies corresponding to predictions from Equation (9).

An expression for the limiting similarity expected between species in competitive trade-off models has been derived by Tilman [12] and May & Nowak [11], which defined the minimum difference that was required in colonization rate, *β*, between two species for the successful invasion of the inferior competitor. We can test the robustness of the predicted limiting similarity to evolutionary dynamics by implementing stochastic simulations of Equation (1) as detailed in the Methods, while allowing for mutation and selection processes to be applied to introduced asexually reproducing individuals along the *β* phenotypic space. Results confirm the evolutionary extinction of all species introduced as they approach the minimum trait threshold *β*_0_ [13] (Fig. 1A), due to the continuous decrease of their densities and subsequent stochastic extinction [28], as was suggested by the results of earlier studies by Kinzig *et al.* [13]. In other words, it appears impossible for a stable community assemblage to evolve given the assumptions underlying Equation (1).

### Defining metapopulation fitness under conditions of competitive uncertainty

We now analyze the adaptive dynamics of a multispecies metacommunity as defined by Calcagno et al.'s [14] extension of Tilman's [12] model:
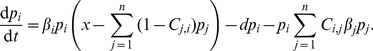
(2)Following Law *et al.* [25] and Calcagno *et al.* [14] we now assume that the outcome of competitive interactions between species is probabilistic, and not strictly deterministic in nature, as defined by the competition function 
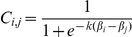
, where *C_i,j_* is the probability that species *j* displaces species *i*. Parameter *k* in the equation indicates the steepness of the competition function, and 1/*k* can be interpreted as the degree of uncertainty in the outcome of competition between species *i* and *j*. High *k* values indicate a strongly deterministic outcome to competitive interactions, while low *k* values describe competitive interactions that are highly probabilistic. Such asymmetric competition between competitors has been associated with co-evolution and stable co-existence between two competitors [25] as well as speciation through deterministic branching in Lotka-Volterra and mechanistic competition models [19], [26].

Using Equation (2) we can define the *per capita* rate of growth *f*_i_ = (1/*p*_i_) · (d*p_i_*/d*t*) as the fitness of species *i* [29]. By setting species *i* to its carrying capacity (*f_i_* = 0), and noting that 1−*C_j,i_* = *C_i,j_*, we get
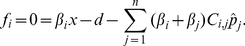
(3)For a system of *n* species we can rewrite the *n* fitness equations as a linear system in matrix form:
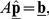
(4)where 
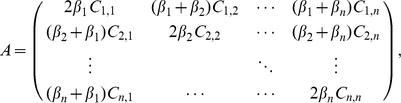


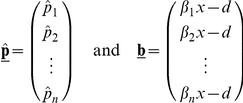
If the matrix *A* is nonsingular (det*A*≠0), then there is a vector of abundances 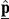 that is a unique solution to the above system of equations. Biologically relevant solutions of 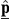 require that the values for all *β_i_* be such that 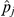
≥0, for all *j*. In contrast to the original competition-colonization model [12], incorporating the competition function *C_i,j_* lead to continuously differentiable fitness functions. We now use the competition function, *C_i,j_*, defined above to study adaptive dynamics of single and multi-species systems.

### Adaptive dynamics of a single species

We start our study of the adaptive dynamics [30]–[32], [29] of a single asexually reproducing species by first considering the fitness, *f_m_*, of a mutant *m* emerging from a single resident species (*species 1*) at carrying capacity:
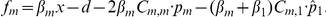
Although we defined fitness here using the per capita growth rate [29], an alternative approach to defining fitness using the total lifetime colonizer output of a single mutant invasive (the metapopulation fitness criteria of Metz and Gyllenberg [33], [34]) can be easily shown to lead to the same adaptive dynamic behaviour.

Since we are interested in the ability of a mutant to invade and establish itself in a resident population when rare we can assume that the invasive/mutant density is very low (*p_m_*≈0), and thus the mutant's fitness equation reduces to 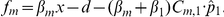
The density 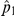 at carrying capacity can be determined from (3) as 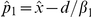. Furthermore, for this solution to be biologically relevant (i.e. 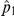
>0) would require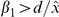.

The gradient of selection, *g*(*β*_1_), when resident *β*_1_ is at its carrying capacity is defined by [32], [29]

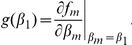



In order to determine our evolutionarily singular points, 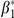, we have to set the selection gradient *g*(*β*_1_) = 0 and solve for *β*_1_. Doing so gives us
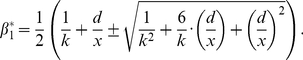
(5)The biologically relevant solution to the above equation is that where 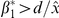. For the singular point 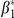 to be an evolutionary attractor the following necessary and sufficient condition would have to be met [32], [29]:
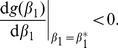
(6)Since it is the case that
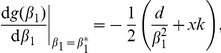
it is clear that the convergent stable condition always holds and that 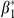 is an evolutionary attractor for all realistic parameter values.

To determine ESS stability a different condition has to be met
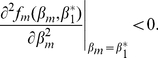
(7)If this condition holds then the singular point 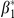 is located at a fitness maximum and neighbouring mutants will be unable to invade, thus preventing evolutionary branching from occurring. Since for a single species 
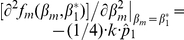
, condition (7) always holds ensuring that 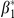 will always be an ESS. If a singular point both attracts nearby strategies and prevents further evolution once they arrive (convergent and ESS stable), as is the case in a single species system, then the singular point is considered a “continuously stable strategy” or CSS [29], [32], [35].

Since for a large range of *k* parameter values (*k*<100) Equation (5) predicts significant differences in trait value between the singular strategy 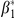 and the minimum threshold for persistence, *β*_0_, it is clear that evolution towards stochastic extinction is no longer inevitable since evolution stops at 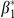 without moving any closer to the low abundance threshold *β*_0_. When the value of *k* decreases 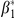 becomes progressively larger, moving further away from the threshold *β*_0_. For large *k* values, we find the solution for the singular positive 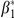 value to be
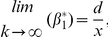
which is also the minimum threshold needed for persistence, 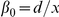. In other words, in the large *k* limit, where the competitive function approximates perfect competitive exclusion, selection will drive species towards the minimum threshold where density becomes arbitrarily close to 0. On a stochastic landscape this will result in an evolution of the population towards eventual extinction – runaway selection leading to self-extinction [28]. This is in fact what was observed with the original metapopulation model with deterministic competitive exclusion. Stochastic simulations demonstrate how moderate *k* values (*k* = 60) allow a single strategy to establish at a singular strategy 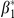, far enough away from the minimum threshold *β*_0_ to avoid extinction (Fig. 1B).

### Co-evolution in multi-species assemblages

We can similarly study the adaptive dynamics of a two species system. However, now we are dealing with two mutants with fitnesses 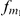, the fitness of the mutant/invasive whose trait value is close to that of the first resident species, and 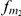, the fitness of the mutant/invasive whose trait value is close to that of the second resident species. Each mutant now competes with both its own resident population and with that of the other resident species. Selection gradients for both populations are:
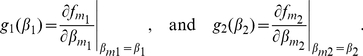
(8)The singular strategy 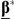 representing a solution to this set of equations is now a vector representing a coalition of strategies, such that 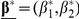. (See Appendix S1 for details). In the limit where the system approximates a strict competitive hierarchy (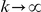), solving for (8) results in the following solution for the singular strategy 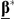:
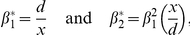
The first expression corresponds to the minimum threshold *β*_0_, and the second to the limiting similarity condition defined by Tilman [12] and May and Nowak [11] when solved for two species. Substituting the first expression into the second expression solves for 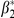 at the minimum threshold *β*_0_. These are precisely the equilibrium values that would be predicted for the special case of perfect competitive exclusion between species.

Using both numerical solutions for (8) and simulation results one can once again observe how a range of *k* values allow both species to avoid evolving to the low abundance threshold of the trait space and hence to possible extinction (See Figure S1 for an example).

The system of nonlinear equations for a *n* species community can be represented in matrix form as follows:
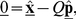
(9)where 
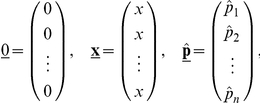
and
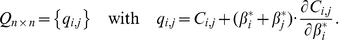
Here 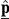 = 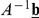 (see (4)). Both numerical solutions and stochastic simulations confirm that introducing competitive uncertainty facilitates diversity build-up (Fig. 1D). In a system open to immigration, species assemble across the entire trait according to their limiting similarity relationship, and avoid aggregating in the high competitive end of the phenotypic trait space (Fig. 1D).

When 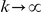, (9) predicts that the evolutionarily singular strategy approaches the minimum abundance threshold, indicating possible evolution towards extinction (Appendix S2). The evolutionary behaviour of the original model (Equation 1) [12] for an *n* species community, as observed in the stochastic simulations, thus appears as a special limiting case of the generalized metapopulation model (Equation (2)). We now detail the importance of competitive uncertainty (*k*) for the assembly of species through both immigration and adaptive radiation.

#### Competitive uncertainty and multi-species assembly

**Figure 2:**
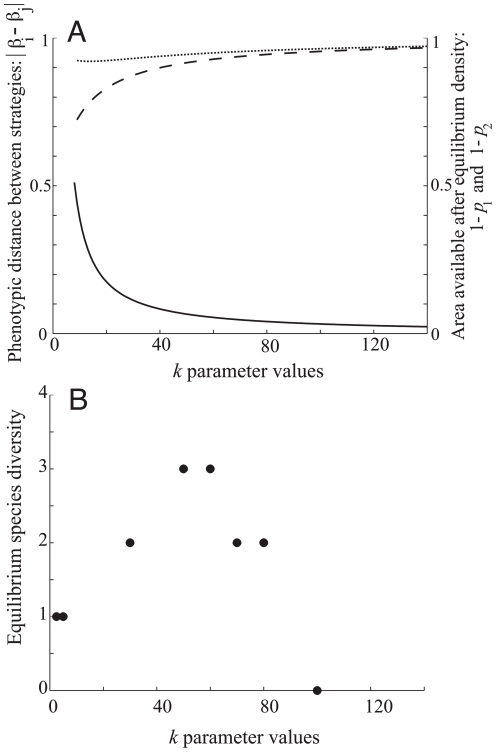
Effects of *k* parameter on phenotypic similarity and diversity. (A) Phenotypic similarity (|*β_i_*−*β_j_*|) between strategies *i* and *j* in a two-species system in relation to *k* (solid line). Area left over after each species reaches equilibrium patch-occupancy (i.e., 1−*p_i_*) in relation to *k* for the superior competitor *p*_1_ (broken line), and the inferior competitor *p*_2_ (dotted line). (B) Equilibrium species diversity in relation to *k* in stochastic simulations. Diversity is measured as the number of ‘stable’ species, i.e. species able to persist or avoid stochastic extinction over >100 000 time steps. Results shown for *d* = 0.6 and 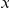 = 0.654.

In metacommunities open to immigration of new species, competitive uncertainty between individuals can promote greater diversity in communities (Fig. 2). The degree of similarity between individual strategies at the singular point 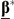 for a two species system (where similarity is measured as the absolute distance in trait value between species) is shown for different *k* values by the solid black curve in Fig. 2A (with similarity being robust to changes in *d*).

Here, in order to incorporate the biologically realistic assumption that there must be some upper-limit in colonizing ability that a species in this system can exhibit, we set an arbitrary finite limit for the maximum *β* value possible of 1. At high *k* values the two species pack in relatively close to each other, as well as close to the minimum threshold *β*_0_. Although the small limiting similarity between species at large *k* values should allow for high biodiversity (Fig. 2A), stochastic extinctions observed in simulations due to extremely low species abundances (dashed and broken lines, Fig. 2A) strongly limit diversity. At the other end of the spectrum (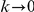) the community of two species collapses into a community of one as the second species is required to evolve beyond the maximum colonizing ability biologically allowed in this model, *β* = 1, in order to persist. The resulting equilibrium diversity over the same range of *k* values can be observed in Fig. 2B. Increasing the biological maximum *β* value allowed increases the number of species that can be packed at intermediated *k* values. Maximum diversity thus corresponds to a balance between limiting similarity and stochastic extinction found at intermediate ranges of *k* imposing within-species variation in competitive ability.

#### Competitive uncertainty and speciation

In metacommunities that are closed to the immigration of new species one important result is the role of the competitive uncertainty parameter *k* for the onset of sympatric speciation through transient phenotypes arising through mutations of non-negligible size. Using deterministic adaptive dynamics we proved with condition (7) that the singular strategy 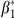 is always an ESS in a one-species system, and therefore no species branching (speciation) can occur. However, in stochastic simulations, as shown in Fig. 1C with mutation steps *μ*>0.001• (1/N) (see Methods for details), the single strategy in the system, as it approaches its final convergent stable state 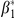, branches into two distinct strategies. This is followed by branching of the second species leading to a three species community, all of whose strategies line up with predictions from (9) for a three species community (*n* = 3). The onset of such stochastic branching is found to be highly sensitive to the degree of competitive uncertainty, *k*.

**Figure 3:**
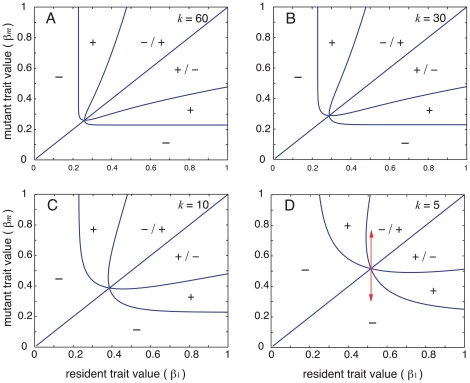
Mutual invasibility plots. Mutual invasibility plots for resident-mutant combinations when (A) *k* = 60, (B) *k* = 30, (C) *k* = 10 and (D) *k* = 5. Plots shown for parameter values *d* = 0.6 and 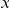 = 0.65. Lines correspond to resident-mutant trait combinations where growth of both strategies is zero. The regions marked by ‘+’ signs indicate resident-mutant trait combinations where either strategy can invade and experience positive growth in the presence of the other strategy, while the ‘−’ signs indicate regions where both experience negative growth in the presence of the other. Mutants experience positive (negative) growth, and residents negative (positive) growth in the trait space marked by +/− (−/+). A vertical line at the singular strategy (the point where all zero-growth isoclines intersect) demonstrates the viability of a mutant arising from a resident at or near the singular strategy (see arrow in plot (D)).

The importance of competitive uncertainty can be understood from a graphical analysis of the deterministic mutual invasibility conditions. Mutual invasibility plots show how small mutations along the vertical (mutant) axis will lead to evolution towards the singular strategy *β* ^*^ (Fig. 3). Drawing a vertical line at this fixed point shows the viability of mutants arising from a resident strategy at this point in trait space [32]. In our metapopulation model condition (7) demonstrates how mutants that are arbitrarily close to this resident strategy will always be in a region of negative growth regardless of the value of *k*, and will thus be unable to invade the system. However in simulations, given that we allow for non-negligible mutation in trait values, and for the associated accumulation of mutants across phenotypic space, the possibility arises for speciation because the phenotypic distance separating mutants from positive fitness becomes smaller as *k* is increased (Fig. 3A: *k* = 60). For small values of *k* the regions of negative fitness around the resident strategy is large and hard to bridge (Fig. 3C: *k* = 10 and Fig. 3D: *k* = 5). Alternatively, for very large *k* values (e.g. *k*≥100), *β* ^*^ is near the minimum threshold *β*_0_ where low abundances of the resident species render the resident population either prone to extinction, or cause the adjacent niche space to occur too close to the resident strategy for the establishment of a distinct species. Only at intermediate levels of competitive uncertainty – this time representing a balance between the need to minimize the distances between niches while maximizing the viability of distinct strains – can one see the possibility of disruptive selection leading to successful speciation. As a result, stochastic simulation results reveal maximum diversity resulting from adaptive radiation for 40<*k*<80 (Fig. 2B). What is important in our results is the presence of evolutionary and ecological constraints that maximize both species packing and speciation for intermediate competitive uncertainty.

## Discussion

Metapopulation theory has served as an important theoretical framework for studying assemblages of species in competitive metacommunities through competition-colonization tradeoffs [10], [12]. Yet, current ecological models are incompatible with the evolutionary assembly of species in competitive communities. We adopt the continuous competition function studied by Calcagno et al. [14], interpreted here as uncertainty in the competitive interactions between species, in order to allow current metapopulation models based on competition-colonization tradeoffs to serve as a framework for studying the evolutionary assembly of communities. Our model allows for the evolution of species packing and abundance distributions. Results show how the degree of uncertainty in competitive interactions between species determines both the extent of biodiversity build-up and its underlying ecological and evolutionary causes. Specifically, intermediate competitive uncertainty, *k*, maximizes biodiversity by (i) striking a balance between species packing and species abundance (i.e. the ability to avoid extinction), and by (ii) balancing the countervailing effects of phenotypic distance and species viability on the chances of disruptive selection.

### Evolutionary consequences of competitive uncertainty: Balancing species extinction vs. species packing

The theoretical assumption underlying most competitive trade-off models is the idea that “differences among species overwhelm variability among individuals, so much so that individual differences can be ignored” [21]. This assumption was key to the development of theories of competition-colonization tradeoff mediated coexistence [10], [12]. In their attempt to test the competition-colonization tradeoff amongst forest trees Clark et al. [21], [22] have suggested that *individual differences* within species may actually be the critical factor weakening competitive exclusion and promoting coexistence and biodiversity. This hypothesis has helped explain how competitively inferior *Acer rubrum* species can coexist with competitively superior *Liriodendron tulipifera* in forest tree communities [22].

Although the effects of intraspecific variation have been recently highlighted as an important factor in structuring natural communities [36]–[38], the presence of such intraspecific variation is not required for interspecific competition to be defined or characterized by competitive uncertainty. For example, a similar weakening of the competition-colonization trade-off has been shown to occur through environmental heterogeneity, which when accounted for, allowed more biologically realistic predictions of species abundance patterns in annual plant assemblages [39].

Incorporating a more probabilistic interpretation of competitive interactions between individuals through a continuous competition function, *C_i,j_*, allows for evolutionary assembly of communities over a range of values for a new parameter, *k*, which signified the degree to which within population variability rendered competition more probabilistic in nature. High *k* values corresponded to situations where populations exhibited little intraspecific variation and hence more deterministic outcomes. Low *k* values signified a high variability within populations for competitive ability, weakening the one-to-one link between competitive and colonizing capability. Furthermore, we were able to demonstrate that in the limit of large *k* values the predictions of our deterministic model converge to those of metapopulation models with strict deterministic exclusion. Predictions of previous metapopulation models [13] are thus a special limiting case of our generalized model.

We also noted how decreasing the value of *k* increases the phenotypic distance between strains, thus decreasing the total number of species within the trait space, while at the same time allowing species to persist or avoid extinction through increased abundances. At increasingly high *k* values the reduction in limiting similarity increases the potential number of available niches in the trait space but at the cost of lowering species abundances to the point where stochastic extinction becomes inevitable. In other words, extreme values of *k* decrease the potential diversity in the community, either through increased probability of extinction or through decreased niche availability. The trade-off between species packing and increased extinction risk associated with *k* means that biodiversity is maximized for intermediate values of competitive uncertainty corresponding to a balance between these two antagonistic processes.

Our approach is also motivated by earlier theoretical studies of mechanistic models based on specific biological systems, such as Jansen & Mulder's [19] study of seed competition in seasonally reproducing organisms with a competition-fecundity trade-off. Similarly, Bonsall & Mangel [20] and Bonsall *et al.* [40] studied trade-offs between competitive ability and such life-history traits as longevity and parasitoid attack rate in models of evolutionary assembly of rockfish (*Sebastes*) communities, and the evolutionary emergence of polymorphism in parasitoid guilds. We were able to use a generalized metapopulation framework to predict species packing and biodiversity build-up through the incorporation of competitive uncertainty. It is because we were able to utilize a standard multi-species modelling framework that the role of competitive uncertainty on the evolutionary assembly of competitive communities is able to have a general interpretation.

### Competitive uncertainty and biodiversity buildup

Our evolutionary model offers a simple measure (*k*) of the degree of uncertainty in competitive interactions. Similar functions to the competition function used here have been used to study the effects of asymmetric competition in allowing the co-evolution and stable coexistence of species pairs [25]. Here we generalize these results to the evolution of whole competitive communities. We more precisely demonstrate how competitive uncertainty allows for the evolution of stable multi-species communities by preventing evolution towards unrealistic packing at the low abundance threshold, and by allowing the equilibrium assemblage to be defined by discrete phenotypic distances between species. As a direct consequence of this generalization, we are able to predict the functional relationship between competitive uncertainty and biodiversity.

By using microcosm experiments to study the outcome of pairwise interspecific interactions it may be possible test whether this degree of competitive uncertainty between species could be quantified, and perhaps whether a corresponding probability kernel – relating the average trait values between pairs of species, and the likelihood of competitive exclusion – could be drawn for a given guild. Experimentally determining which species are then capable of being assembled together into stable communities would provide an important first step in testing whether the mechanism outlined here plays an important role in allowing biodiversity build-up in natural systems.

### Competitive uncertainty and sympatric speciation: The role of transient variation arising from non-negligible mutation sizes

Our results reveal conditions allowing competitive uncertainty to explain the occurrence of sympatric speciation and adaptive radiation in metacommunities. The emergence of biodiversity through sympatric speciation or evolutionary branching has been investigated using several models [19], [20], [26], [40]. However, in all such studies speciation or evolutionary branching was explored as a deterministic process arising from violations of ESS stability at various fixed points in trait space. Our analysis demonstrates that such a deterministic branching process is not permitted in our metacommunity model where speciation requires the introduction of mutations of non-negligible size, capable of creating enough transient variation to bridge the limiting similarity in trait distance required for successful invasion and coexistence.

Such interaction between deterministic and stochastic processes to explain the maintenance and structure of communities within adaptive dynamics frameworks has been revealed using Lotka-Volterra competition [41]. It has been used to explore the possibility of sympatric speciation under conditions of environmental, demographic and resource stochasticity [42], [43]. What is important here is the driving role of competitive uncertainty, *k*, on the occurrence of sympatric speciation in the presence of transient variation arising from non-negligible mutation sizes. Our analysis demonstrates how *k* affects the distance in trait space between adjacent potential niches, allowing phenotypic variation to lead to disruptive selection. Low *k* values result in distances between niches that are too large to be crossed by transient types, while high *k* values result in either no differentiation, or in stochastic extinction. As with the effects of competitive uncertainty on species packing, intermediate *k* values are key in explaining adaptive radiation in competitive communities with competition-colonization tradeoffs.

These results are of particular relevance given the growing recognition that both ecological and evolutionary theory's focus on the long-term, or the asymptotic behaviour of systems may be undermining an appreciation of the more transient dynamics or processes that are involved in structuring natural communities [44]. What is striking in our results is the impact of uncertainty in an ecological process (i.e. competition) on the onset of an evolutionary process like speciation. Future work should generalize results presented here to sexually reproducing populations and explore the importance of competitive uncertainty for the evolution of assortative mating and sympatric speciation [29].

### Conclusions

Incorporating a continuous competition function that acknowledges the probabilistic nature of competitive exclusion between species was found to enable standard metapopulation models to predict the evolutionary assemblage of competitive communities. Intermediate values of competitive uncertainty maximize biodiversity in open metacommunities, and allow sympatric speciation and adaptive radiation through the persistence of transient phenotypes in metacommunities that are closed to immigration. The degree of competitive uncertainty has a biological basis in the intra-specific variability of competitive ability present in natural populations. We have demonstrated how such a probabilistic interpretation of competitive interactions can allow a generalized metapopulation theory to serve as a viable framework for studying the evolutionary assembly of competitive guilds and the evolution of life-history tradeoffs.

## Methods

### Stochastic simulations

Simulations provided a stochastic and individual based model version of the mean-field equation (2). Simulations were conducted on a 65,536 (256×256) cell lattice with periodic boundary conditions, where individual cells represented habitat patches, and where each available habitat site in the landscape could be potentially occupied by at most one individual at a time. Since in Equation (2), *x* represents the fraction of available habitat, the number of cells in the lattice that were made available for colonization or occupancy by individual organisms during simulations was simply 65,536*x*.

#### Community dynamics

All the cells in the lattice were updated asynchronously at each time-step to approximate a continuous time process. Although we implemented continuous time dynamics, we refer to 1 time step as 65,536 randomly selected cell updates. Updating cells involved allowing for colonization of new sites, and for mortality within already occupied sites. Individual mortality in our model was a result of death occurring in an occupied cell at a rate *d*, or as the result of competitive displacement by a newly arriving colonizer. Colonization involved individuals in the landscape dispersing new colonizers to randomly selected sites at a rate *β*. The success of a new colonizer establishing itself depended on whether the new site was empty (in which case, the probability of establishing was equal to 1), or whether it was already occupied (in which case the probability of competitively excluding the current resident and establishing itself was *C_i,j_* in Equation (2)).

#### Evolutionary dynamics

Each possible ‘species’ or strain in our model was defined by a single trait value between 0 and 1 corresponding to colonizing ability, *β*. The phenotype space, or trait gradient, between 0 and 1 was evenly divided into *N* values (*N* = 1000 for all simulations). Mutation size along the trait gradient was implemented using a Poisson distribution defined by *μ*, the average distance in trait value away from the parent's phenotype for every successful bout of reproduction. Using this distribution we determined the number of discrete steps away from the parent along the trait gradient a new colonizer's phenotype will be after every bout of reproduction or colonization. In our simulations *μ* ranged from 0.0095*(1/N) (high average mutation) to 0.001*(1/N) (low average mutation).

In simulations where immigration of new species was allowed, species were introduced from the low competitive/high colonizing end of the trait gradient at regular intervals of 50 000 time-steps.

## Supporting Information

Appendix S1 **Adaptive dynamics of a two-species system.** Solution for the singular strategies of two-species system.
(DOCX)

Appendix S2 **Evolutionarily singular strategy for an** ***n*** **-species system.** Demonstration of how the joint singular strategy of an n-species system will approach the zero-abundance threshold for large *k* values.
(DOCX)

Figure S1
**Solutions for coevolving species pair.** Graphical depiction of the possible solutions for the system of nonlinear equations described by (A3a) and (A3b) in Appendix S1 when *k* = 10, *d* = 0.6 and habitat availability of 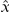 = 0.654. Solutions to the system of equations are represented by the intersection of the curves described by the two equations. Each possible solution is a set of paired values of *β*_1_ and *β*_2_ representing a coalition of strategies. In the example shown here only points *a* and *b* represent biologically realistic solutions. Points *c* and *d* give values for *β*_1_ and *β*_2_ that entail biologically unrealistic abundance values – i.e. negative abundances. Since points *a* and *b* are equivalent solutions, with just the values for the two strategies reversed, there only exists one possible singular strategy, *β_a_* ^*^ = (*β*_1_^*^, *β*_2_^*^), in the example depicted here. The solution for point *a* estimated using a numerical algorithm solver was found to be *β* ^*^ = (*β*_1_^*^, *β*_2_^*^)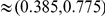.(EPS)
