# Supplementary material for: Competition-Colonization Trade-Offs, Competitive Uncertainty, and the Evolutionary Assembly of Species
Source: PLoS One. 2012 Mar 20;7(3):e33566. doi: 10.1371/journal.pone.0033566 (PMC3308991; doi:10.1371/journal.pone.0033566)
Supplement: Appendix S1 — Adaptive dynamics of a two-species system. Solution for the singular strategies of two-species system. (DOCX) [file pone.0033566.s001.docx]

### Appendix S1:

### Adaptive dynamics of a two-species system

Below we demonstrate the adaptive dynamics for a competitive two-species metapopulation model in a manner similar to the single species system. For two species we have the following fitness functions when both are at carrying capacity:

(A1a)

(A2b)

The system of equations represented by Equation (4), for *n*=2, can be used to determine the explicit expressions for the equilibrium densities of$\hat{p}_{1}$and$\hat{p}_{2}$.

We now will consider the fitness of mutants arising from both resident species *1* and *2*. First we designate $f_{m_{1}}$as the fitness of the mutant/invasive whose trait value is close to that of *species 1*, and $f_{m_{2}}$as the fitness of the mutant/invasive whose trait value is close to that of *species 2*. $f_{m_{1}}$and $f_{m_{2}}$are defined by the following equations:

Each mutant now not competes with both its own resident population and with that of the other resident. The gradients of selection for the two populations are:

$g_{1}(\beta_{1})=\left.\frac{\partial f_{m_{1}}}{\partial\beta_{m_{1}}}\right|_{\beta_{m1}=\beta_{1}}$, and $g_{2}(\beta_{2})=\left.\frac{\partial f_{m_{2}}}{\partial\beta_{m_{2}}}\right|_{\beta_{m2}=\beta_{2}}$. (A2)

The singular strategy $\underline{\mathbf{\beta}}^{*}$ now represents a coalition of strategies, such that $\underline{\mathbf{\beta}^{*}}=(\beta_{1}^{*},\beta_{2}^{*})$. To find the singular strategy $\underline{\mathbf{\beta}^{*}}=(\beta_{1}^{*},\beta_{2}^{*})$for a two species system we set both the *g*_1_(*β*_1_) and *g*_2_(*β*_2_) to 0, and solve the resulting system of nonlinear equations in order to find the solutions for trait values$\beta_{1}^{*}$and$\beta_{2}^{*}$:

, (A3a)

and

, (A3b)

where$\hat{p}_{1}$and $\hat{p}_{2}$were defined previously, and where

$\frac{\partial C_{1,2}}{\partial\beta_{1}}=\frac{\partial C_{2,1}}{\partial\beta_{2}}=k\left(e^{1/2k(\beta_{1-}-\beta_{2})}+e^{-1/2k(\beta_{1-}-\beta_{2})}\right)^{-2}$.

The solution to this set of nonlinear equations,$\underline{\mathbf{\beta}^{*}}=(\beta_{1}^{*},\beta_{2}^{*})$, lies on the intersection of the two curves represented by both equations (Fig. S1). Although there is no obvious closed-form solution to the system of equations in the manner of the one species system, solutions can be readily estimated using numerical methods.

The method for determining ESS stability for a two (or more) species system becomes much more complicated than for a one species system. The general condition described by Equation (8) can be used to determine the ESS stability of each species separately only if the other species in the pair is assumed to already be ESS stable at the singular point and thus not prone to branching [26]. Determining convergence stability to ascertain if the vector $\underline{\mathbf{\beta}}^{*}$ represents an evolutionary attractor for the community may be done by determining whether the real parts of the eigenvalues of the Jacobian matrix of the system, **J**, are negative

$\mathbf{J}=\left(\begin{matrix} m_{1}\left(\beta_{1}^{*}\right)\frac{\partial g_{1}(\beta_{1},\beta_{2})}{\partial\beta_{1}} & m_{1}(\beta_{1}^{*})\frac{\partial g_{1}(\beta_{1},\beta_{2})}{\partial\beta_{2}} \\ m_{2}\left(\beta_{2}^{*}\right)\frac{\partial g_{2}(\beta_{1},\beta_{2})}{\partial\beta_{1}} & m_{2}(\beta_{2}^{*})\frac{\partial g_{2}(\beta_{1},\beta_{2})}{\partial\beta_{2}} \end{matrix}\right)_{\beta_{1}^{*},\beta_{2}^{*}}$,

where *m*_1_(*β*_1_) and *m*_2_(*β*_2_) are quantities describing the effects the mutation processes has on the speed of evolution in the two species when at the given trait values *β*_1_ and *β*_2_ [29].
