# Supplementary material for: Competition-Colonization Trade-Offs, Competitive Uncertainty, and the Evolutionary Assembly of Species
Source: PLoS One. 2012 Mar 20;7(3):e33566. doi: 10.1371/journal.pone.0033566 (PMC3308991; doi:10.1371/journal.pone.0033566)
Supplement: Appendix S2 — Evolutionarily singular strategy for an n -species system. Demonstration of how the joint singular strategy of an n-species system will approach the zero-abundance threshold for large k values. (DOCX) [file pone.0033566.s002.docx]

### Appendix S2

We proceed below to demonstrate that *all* species in an arbitrary *n* species system will have a singular strategy at (and thus, can potentially evolve towards) the minimum threshold required for persistence, *β*_0_, when competitive exclusion is strictly deterministic (i.e. $k\to\infty$). In order to determine the singular strategy of an *n* species community, $\underline{\mathbf{\beta}}^{*}=\left(\beta_{1}^{*},\ldots,\beta_{n}^{*}\right)$, when *k* is arbitrarily large we take the limit as $k\to\infty$ for the system of equations represented by (9), here represented by the *r*^th^ equation:

.

For any arbitrary species *r* the limit reduces to:

(B.1)

The expression for the equilibrium abundance of the *r*^th^ species,$\hat{p}_{r}$, can be found by solving equation (2) when d*p_i_*/d*t* = 0, determining the expression for the first species $\hat{p}_{1}$, and then substituting it in to the expression for the abundance of the second species $\hat{p}_{2}$. A process of sequential substitutions can be continued until an expression is determined for the *r*^th^ species. Alternatively, we can solve for the *r*^th^ species abundance using Cramer’s rule for the linear system represented by equation (4): $\hat{p}_{r}=\frac{\det A_{r}}{\det A}$, where *A_r_* is the matrix *A* with its *r*^th^ column vector replaced with vector $\underline{\mathbf{b}}$ from (4). We thus get

$\begin{matrix} \lim\\ k\to\infty\end{matrix}\text{ }\hat{p}_{r}=\frac{\begin{matrix} \lim\\ k\to\infty\end{matrix}\text{ }\det A_{r}}{\begin{matrix} \lim\\ k\to\infty\end{matrix}\text{ }\det A}\text{ }(\text{provided }\lim_{k\to\infty} \text{ }\det A\neq0)$ (B.2)

The expression in the denominator can be shown to reduce to $\begin{matrix} \lim\\ k\to\infty\end{matrix}\det A=\beta_{1}\cdot\beta_{2}\cdots\beta_{n}$. For the numerator, by sequentially taking the Laplace expansion starting on the last column of *A_r_* one can show the solution to be , where $\Omega_{r}$ represents the expression:

. (B.3)

Substituting the numerator and denominator into (B.2) gives us

. (B.4)

When we substitute (B.4) into (B.1) we can see the linear system represented by (9) reduces to

(B.5)

Starting with the first species then sequentially solving for *β* and substituting the values of each *β* into the expression for the next species, it can be readily seen (by inspection of (B.3)) that for all species equation (B.5) reduces to $(\beta_{r}x-d)$= 0, or alternatively *β*_r_ = *d*/*x*, which is the expression for *β*_0_, the minimum or zero abundance threshold. That is, the evolutionarily singular strategy $\underline{\mathbf{\beta}}^{*}\to\left(\frac{d}{x},\frac{d}{x},\ldots,\frac{d}{x}\right)$ as $k\to\infty$; therefore the singular strategy of all species is at the zero abundance threshold when competitive exclusion is the outcome of a strictly deterministic competitive process, which under condition of convergence stability implies that all species will evolve to extinction.
